# Roles of cofactors and chromatin accessibility in Hox protein target specificity

**DOI:** 10.1186/s13072-015-0049-x

**Published:** 2016-01-08

**Authors:** Ching Yew Beh, Sherif El-Sharnouby, Aikaterini Chatzipli, Steven Russell, Siew Woh Choo, Robert White

**Affiliations:** Department of Oral Biology and Biomedical Sciences, Faculty of Dentistry, University of Malaya, 50603 Kuala Lumpur, Malaysia; Department of Physiology, Development and Neuroscience, University of Cambridge, Downing Street, Cambridge, CB2 3DY UK; Department of Genetics, University of Cambridge, Downing Street, Cambridge, CB2 3EH UK; Cambridge Systems Biology Centre, University of Cambridge, Tennis Court Road, Cambridge, CB2 1QR UK

**Keywords:** Hox proteins, Chromatin accessibility, Transcription factor

## Abstract

**Background:**

The regulation of specific target genes by transcription factors is central to our understanding of gene network control in developmental and physiological processes yet how target specificity is achieved is still poorly understood. This is well illustrated by the Hox family of transcription factors as their limited in vitro DNA-binding specificity contrasts with their clear in vivo functional specificity.

**Results:**

We generated genome-wide binding profiles for three Hox proteins, Ubx, Abd-A and Abd-B, following transient expression in *Drosophila* Kc167 cells, revealing clear target specificity and a striking influence of chromatin accessibility. In the absence of the TALE class homeodomain cofactors Exd and Hth, Ubx and Abd-A bind at a very similar set of target sites in accessible chromatin, whereas Abd-B binds at an additional specific set of targets. Provision of Hox cofactors Exd and Hth considerably modifies the Ubx genome-wide binding profile enabling Ubx to bind at an additional novel set of targets. Both the Abd-B specific targets and the cofactor-dependent Ubx targets are in chromatin that is relatively DNase1 inaccessible prior to the expression of Hox proteins/Hox cofactors.

**Conclusions:**

Our experiments demonstrate a strong role for chromatin accessibility in Hox protein binding and suggest that Hox protein competition with nucleosomes has a major role in Hox protein target specificity in vivo.

**Electronic supplementary material:**

The online version of this article (doi:10.1186/s13072-015-0049-x) contains supplementary material, which is available to authorized users.

## Background

The control of specific sets of target genes by transcription factors is central to our understanding of the gene networks regulating development, physiological responses and disease processes. However, many questions remain concerning how transcription factors identify their specific targets in the genome. In particular, many transcription factors bind to short degenerate sequence motifs, which occur at high frequency in the genome, and it is unclear how their regulatory function is restricted to specific sets of targets. Hox proteins are key developmental regulators that illustrate this issue well. They bind to short AT-rich motifs with individual members of the Hox family exhibiting very similar in vitro DNA-binding preferences (reviewed in [1]). In contrast, different Hox proteins show clear in vivo functional specificity by regulating the development of very different segmental morphologies (reviewed in [2]). The discrepancy between the relative lack of in vitro DNA-binding specificity and the clear in vivo developmental specificity is still unresolved. The identification of Hox cofactors, proteins that interact with Hox proteins and enhance their DNA-binding specificity, provides a partial explanation. The Three Amino acid Loop Extension (TALE) homeodomain cofactors, Extradenticle (Exd) and Homothorax (Hth) in *Drosophila* and their vertebrate Pbx/Meis homologues, increase the specificity of Hox binding in vitro [3–5] and contribute to in vivo Hox specificity (reviewed in [1]). These Hox cofactors have been shown to engage in tripartite Hox/Exd/Hth (Hox/Pbx/Meis) complexes that enable Hox functional specificity in vivo at particular target sites [6–8]. In addition, interaction between Hox proteins and these cofactors differentially affects the binding preferences of Hox proteins enabling the emergence of “latent specificity” of Hox binding in the context of the Hox-cofactor complex [9]. However, in some situations Hox proteins act in the absence of Exd/Hth, for example in the classic transformation of the *Drosophila* wing blade primordium to haltere by the Hox protein Ultrabithorax (Ubx) [10]. Although the in vivo study of endogenous Hox target gene regulatory elements has provided insight into the roles of cofactors at specific targets, relatively few target sites have been studied and there is no clear general understanding of the role of cofactors in Hox specificity; indeed at some regulatory elements the cofactors appear to be required for specific functional activity, gene activation rather than repression, and not for DNA-binding specificity [11].

In general, although there is a wealth of in vitro data on Hox specificity [12, 13], how these extrapolate to the in vivo situation is currently unclear. Genome-wide studies in *Drosophila* tissues have investigated Hox protein and cofactor binding and have identified Hox binding sites and target genes [14–16]. These in vivo studies also suggest an important role for chromatin accessibility in determining where in the genome Hox proteins bind, supporting the view that chromatin accessibility generally plays a key role in transcription factor target specificity [17]. However, for the detailed analysis of Hox binding specificity, in vivo tissues have the disadvantage of cellular heterogeneity with different cells having different chromatin states and cofactor expression. Here we have investigated Hox binding specificity in a *Drosophila* cell line that is relatively homogeneous, facilitating a rigorous comparative analysis of the binding of different Hox proteins in the same genomic background and under the same conditions. Using carefully controlled Hox protein expression in Kc167 cells we show that Hox proteins exhibit clear target specificity in the absence of the Exd and Hth cofactors. These cofactors can nevertheless play a key role in Hox protein binding and we demonstrate their effect on the genome-wide target specificity of Ubx. Our experiments also provide further evidence of the strong role chromatin accessibility plays in Hox binding and suggest that Hox competition with nucleosomes has a major role in Hox target specificity in vivo.

## Results

### Specific Hox protein binding in Kc167 cells

To compare the binding profiles of different Hox proteins, we used transient transfection of *Drosophila* Kc167 cells with inducible expression constructs producing Hox-GFP fusion proteins and generated genome-wide binding profiles using Chromatin Immunoprecipitation followed by high-throughput sequencing (ChIP-Seq). Kc167 cells do not express Hox proteins endogenously and also lack functional Exd and Hth cofactors; although Exd is expressed, in the absence of Hth it is cytoplasmic. In order to ensure that the Hox binding profiles generated by our experiments are as comparable as possible: (1) we processed the different samples in parallel using cells taken from the same mother-culture, (2) we sorted the transfected cells 4 h after expression induction using a Fluorescence-Activated Cell Sorter (FACS) to select cells expressing the same level of Hox-GFP fusion protein and (3) we used the same anti-GFP antibody for all ChIP assays (Additional file 1: Figure S1, S2). We confirmed that the Hox-GFP expression level is close to the physiological range. Estimates of the number of homeodomain protein molecules per nucleus range from 20,000 to 50,000 [18–21]. We estimate that the Ubx-GFP expressing cell population selected by FACS has expression levels from 38,000 to 74,000 Ubx-GFP molecules per cell (see “Methods”).

**Fig. 1:**
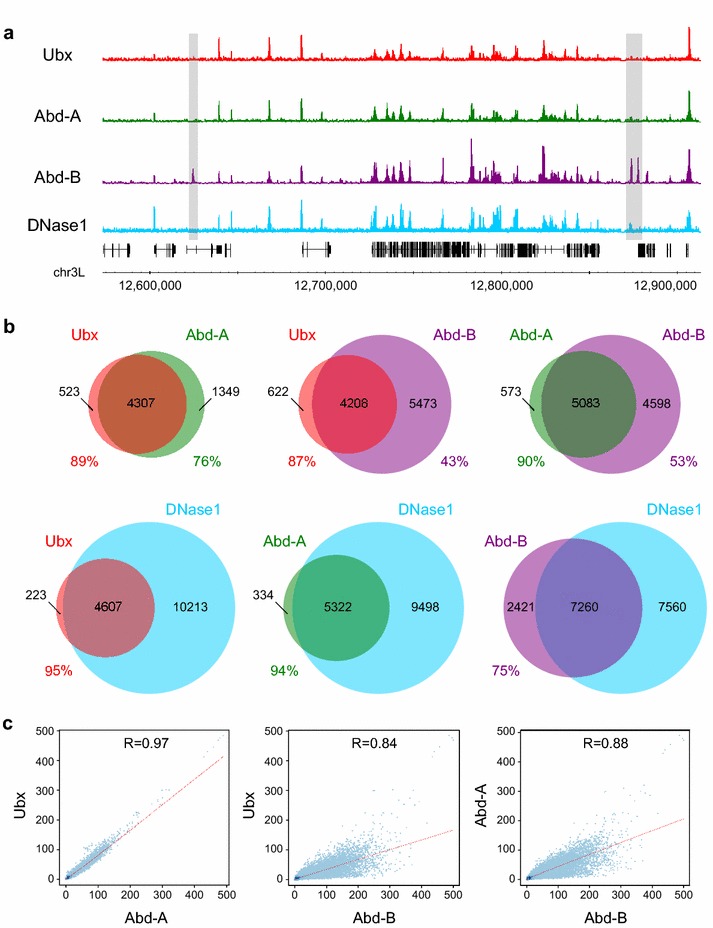
Hox protein binding and chromatin accessibility in Kc167 cells. **a** Comparison of binding profiles of the three Bithorax complex Hox proteins (Ubx, Abd-A and Abd-B) and DNase1 accessibility in Kc167 cells. Examples of Abd-B specific peaks are highlighted in *grey*. **b** Venn diagrams showing overlap analysis of binding peaks. Hox peaks are q-value 1e−10 and DNase1 peaks are q-value 1e−2. Percentage overlap is indicated. The overlap of Abd-A and Ubx is reinforced by stringent versus relaxed analysis (i.e. overlap of q-value 1e−10 peaks with q-value 1e−2 peaks); for example, Abd-A stringent almost completely overlaps Ubx relaxed (99.6 %), whereas Abd-B stringent only has 75 % overlap with Ubx relaxed. **c** *Scatter plots* showing Pearson’s correlations between the Hox protein binding profiles based on binding score per 1 kb window. The Ubx and Abd-A profiles are highly correlated, while the correlations with Abd-B are lower and the binding scores more scattered

We compared the binding of the three Bithorax complex Hox proteins; Ubx, Abdominal-A (Abd-A) and Abdominal-B (Abd-B), with a visual inspection of the binding profiles suggesting highly similar Ubx and Abd-A binding. In contrast, while Abd-B largely binds at the same sites as Ubx and Abd-A, it also clearly binds at a considerable number of additional sites (Fig. 1a). To quantify this we carried out overlap analysis of the binding peaks and found, for example, that 89 % of Ubx peaks overlap with Abd-A peaks (Fig. 1b). In contrast, while 90 % of Abd-A peaks overlap with Abd-B peaks, Abd-B clearly binds at additional sites as only 53 % of Abd-B peaks overlap Abd-A peaks (Fig. 1b). Our observations are further supported by Pearson’s correlations between the binding scores of different Hox proteins (Fig. 1c). We conclude that Hox proteins exhibit clear target specificity when binding to Kc167 cell chromatin.

**Fig. 2:**
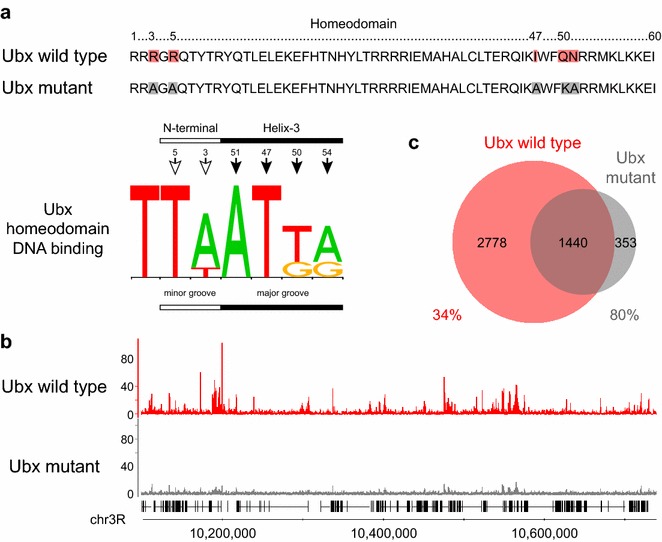
Binding of the Hox-GFP fusion proteins in Kc167 cells is mainly dependent on direct interaction with DNA. **a** The homeodomain sequence of Ubx protein. The Arg3, Arg5, Ile47, Gln50 and Asn51 residues, mediating DNA contacts in the major and minor groves (red in Ubx wild type) were mutated to Ala3, Ala5, Ala47, Lys50 and Ala51 (*grey* in Ubx mutant), abolishing the ability to bind DNA. The Ubx motif sequence logo is from the JASPAR database (MA0094.2). **b** Comparison of the binding profiles (with fragment pileup signal normalized per million reads) of wild type and mutant Ubx (Experiment 2), with the mutant showing a strong reduction in binding. **c** Venn diagram showing the overlap of binding peaks (q-value 1e−10) between Ubx wild type and mutant. About 66 % of Ubx wild type peaks do not overlap with Ubx mutant peaks

To investigate how much of the observed Hox binding to chromatin is dependent on direct DNA binding, we compared the binding of the wild type Ubx protein with a Ubx protein carrying mutations in homeodomain residues mediating DNA contact (Arg3, Arg5, Ile47, Gln50 and Asn51 of the homeodomain; Fig. 2a); these mutations have been shown to abolish DNA-binding in vitro [22, 23]. The binding profiles of wild type and mutant Ubx clearly demonstrate that mutation of DNA contact residues produces a strong reduction in binding (Fig. 2b). We observed a reduction in binding peaks from 4218 with wild type Ubx, to 1793 with mutant Ubx, with approximately 66 % of wild type Ubx peaks showing no overlap with mutant Ubx peaks (Fig. 2c). In addition, the DNA sequences underlying mutant Ubx binding peaks are not enriched for Hox Position Weight Matrices (PWMs; see below and Fig. 4b). Thus the majority of the Hox-GFP binding we observe is likely to depend on direct interaction with DNA rather than indirect binding through, for example, protein–protein interactions.

### Ubx binds at additional sites in the presence of Exd and Hth

**Fig. 3:**
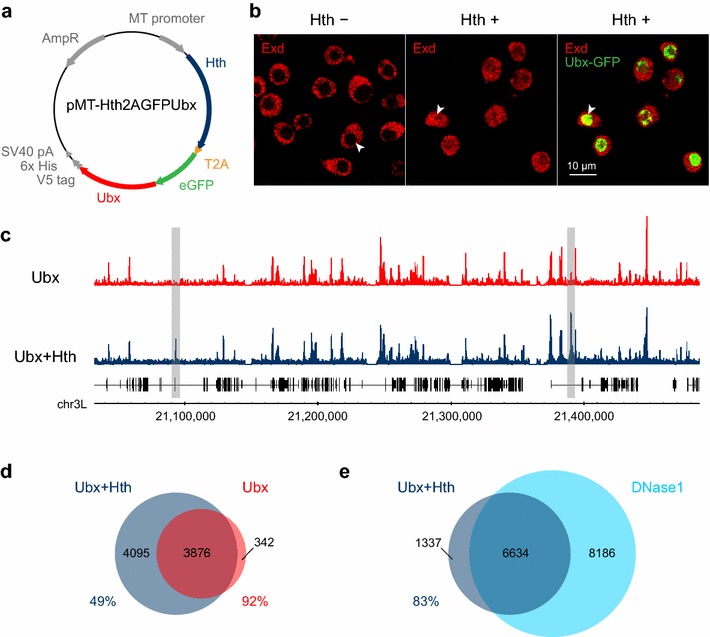
Hox cofactors Exd and Hth alter the binding profile of Ubx. **a** The pMT-Hth2AGFPUbx bicistronic expression vector used to co-express Hth and Ubx-GFP in Kc167 cells. The construct contains the *Drosophila* metallothionein (MT) promoter, Hth cDNA isoform A, *Thosea asigna* 2A self-cleaving peptide (T2A), enhanced Green Fluorescent Protein (eGFP) fused to Ubx cDNA isoform E, C-terminal peptide (containing V5 and polyhistidine tags and SV40 polyadenylation signal), and an ampicillin resistance gene. **b** Exd immunolabelling (*red*) was used to confirm that transfection of Kc167 cells with pMT-Hth2AGFPUbx results in the expression of functional Hth with Hth-dependant recruitment of Exd into the nucleus. *Left*: In non-transfected cells (Hth−), Exd is excluded from the nucleus (*arrowhead*). *Middle*: In transfected cells (Hth+), Hth induces nuclear accumulation of Exd protein (arrowhead). Right: Same as ‘Middle’ but also showing Ubx-GFP (*green*). Hth +/− cells were separated by FACS following transfection (Additional file 1: Figure S1). **c** Comparison of binding profiles of Ubx and Ubx in the presence of Hth (Experiment 2). Examples of cofactor-dependent binding are highlighted in *grey*. **d** Venn diagram showing the overlap of binding peaks (q-value 1e–10) between Ubx and Ubx + Hth. 51 % of the Ubx + Hth peaks are novel. **e** Venn diagram showing the overlap of binding peaks between Ubx + Hth and DNase1 (q-value 1e–10 for Ubx + Hth and 1e–2 for DNase1). 17 % of the Ubx + Hth peaks are in DNase1 inaccessible chromatin

To test the role the Hox cofactors Exd and Hth play in Hox binding, we transfected Kc167 cells with a bicistronic construct expressing the Ubx-GFP fusion together with Hth (Fig. 3a) and assayed Ubx binding by ChIP-Seq. As shown in Fig. 3b, although Kc167 cells express Exd, it is exclusively cytoplasmic due to the absence of Hth. The provision of Hth enables the nuclear accumulation of Exd and thus nuclear availability of both cofactors. In comparison with Ubx alone, the expression of Ubx in conjunction with Hth leads to a considerable increase in Ubx binding, with over 4000 high stringency (q-value 1e−10) additional binding peaks (Fig. 3c, d). This clearly demonstrates that the Exd and Hth cofactors can change the binding specificity of Ubx and enable binding at a large number of new sites.

### Hox protein binding and chromatin accessibility

In our previous analysis of genome-wide Ubx and Hth binding in *Drosophila* embryos and imaginal discs, we suggested that the similarity in the Ubx and Hth binding profiles and the exclusion of Ubx and Hth binding from repressed chromatin indicated a major role for chromatin accessibility in governing the binding of these transcription factors [14]. Genome-wide chromatin accessibility has been mapped in the Kc167 cell line by DNase1 profiling [24], and comparing the DNase1 profile to the Hox binding profiles reveals a very strong association between binding and accessibility (Fig. 1a). Overlap analysis (Fig. 1b) indicates that virtually all of the Ubx and Abd-A binding is in pre-existing DNase1 accessible chromatin; for example 95 % of Ubx peaks overlap DNase1 peaks. For Abd-B binding, there is considerable overlap with accessible chromatin, with 75 % of Abd-B peaks overlapping DNase1 peaks. However, in this case we also observe a considerable portion of the binding (25 %) that does not overlap DNase1 peaks. This indicates that Abd-B binds to regions whose accessibility is low prior to the expression of Abd-B, suggesting that Abd-B is able to compete with nucleosomes. For Ubx binding in the presence of Hth (Fig. 3e), the situation is similar to Abd-B; there is considerable overlap with accessible chromatin, with 83 % of Ubx + Hth peaks overlapping DNase1 peaks, and the remaining 17 % of binding occurring in DNase1 inaccessible chromatin.

### Binding motif analysis

**Fig. 4:**
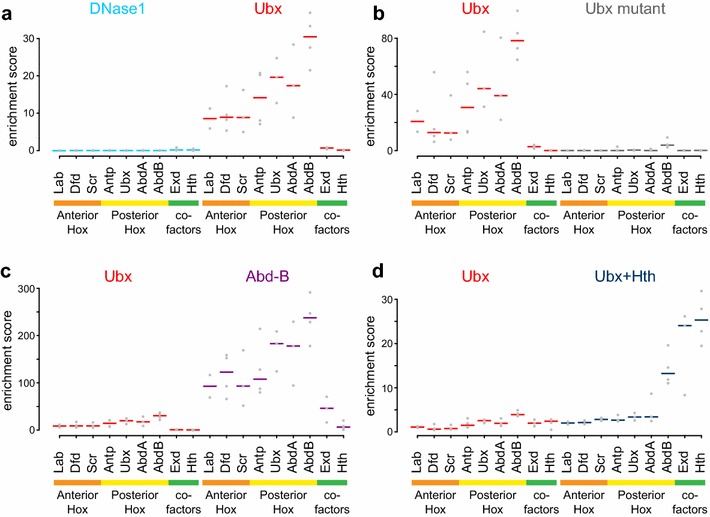
Motif enrichment analysis of the DNA sequences underpinning the binding profiles. Motif enrichment analysis was performed using PWMEnrich for the Hox and Hox cofactor PWM motifs in the MotifDb database. Enrichment scores [log_10_(1/p-value)] for individual motifs are indicated (*grey dots*) together with median for each motif set (*coloured bar*). **a** Comparing motif enrichments for DNase1 (1000 random q-value 1e–2 peaks) and Ubx (1000 q-value 1e–2 peaks with highest Ubx versus DNase1 differential signal; Experiment 1) reveals that DNase1 peaks in general are not enriched for Hox PWMs, whereas Ubx peaks show clear enrichment with the posterior Hox gene PWMs showing the highest enrichment. Little or no enrichment is observed for Hox cofactor PWMs. **b** Comparing Ubx wild type (1000 q-value 1e−10 peaks with highest Ubx versus DNase1 differential signal; Experiment 2) and Ubx mutant (1000 q-value 1e−10 peaks with highest Ubx mutant versus DNase1 differential signal), reveals lack of Hox and Hox cofactor PWM enrichment in Ubx mutant peaks. **c** Comparing Ubx (1000 q-value 1e–2 peaks with highest Ubx versus DNase1 differential signal; Experiment 1) and Abd-B (1000 q-value 1e–2 peaks with highest Abd-B versus DNase1 differential signal), demonstrates that Abd-B peaks have markedly higher enrichments for Hox PWMs but otherwise a similar enrichment profile (cf. Ubx in **a**). **d** Comparing Ubx (328 random q-value 1e–2 peaks common to Ubx Experiment 2 and Ubx + Hth) and Ubx + Hth (328 q-value 1e–2 peaks specific to Ubx + Hth); in the presence of Exd and Hth, the enrichment of Exd and Hth PWMs in the Ubx peaks is increased and the enrichment of Hox PWMs is shifted towards a greater relative preference for Abd-B motifs

To understand the DNA sequences underpinning the Hox binding profiles we performed motif enrichment analysis using PWMs for members of the *Drosophila* Hox family in the MotifDb database. Since the Ubx and Abd-A binding profiles strongly mirror the chromatin accessibility profile, we first examined the DNase1 peaks and found they do not show enrichment for Hox PWMs (Fig. 4a). Initial analysis of Ubx peaks revealed modest enrichment for Hox PWMs. However, the relative level of Ubx versus DNase1 signal varies for different Ubx peaks, so we decided to focus our analysis on the Ubx peaks that show the highest differential between Hox binding and DNase1 accessibility. As shown in Fig. 4a, these peaks show clear enrichment for Hox PWMs. All the Hox PWMs show some enrichment, although the enrichment scores for the posterior Hox PWMs (Antp, Ubx, Abd-A and Abd-B) are higher than for the anterior Hox PWMs (Lab, Dfd and Scr). Interestingly, Abd-B motifs, rather than Ubx motifs, show the highest enrichment. The PWMs for the Hox cofactors show little if any enrichment, in agreement with the fact that these proteins are not functional in Kc167 cells. As described above, the peaks associated with the mutant Ubx protein show no enrichment for Hox PWMs (Fig. 4b). We interpret these data to indicate that, although the Ubx profile closely mirrors chromatin accessibility, Ubx does not bind non-specifically but rather shows clear specificity for Hox PWM motifs within accessible chromatin.

This view is supported by the lack of Hox PWMs in DNase1 accessible regions that show no Ubx binding. For example, the Abd-B PWM “AbdB_SOLEXA_FBgn0000015” is highly enriched in the peaks that show higher Ubx versus DNase1 signal (17th out of the 709 motifs in the MotifDb database, p = 4.8e−34). For the general class of Ubx peaks that overlap DNase1 accessible regions, this Abd-B PWM is still enriched but its ranking drops to 61st (p = 3.3e−16). However, in the DNase1 regions with no Ubx binding, this motif is ranked bottom of the list (p = 1). This indicates that we do not detect Ubx binding in accessible chromatin that lacks Hox PWMs.

Comparing Abd-B peaks with Ubx peaks, again examining peaks with the highest differential between Hox binding and DNase1 accessibility, we find that Abd-B peaks show higher enrichment for Hox PWMs (Fig. 4c). The relative enrichments of the different Hox PWMs show a similar pattern to the Ubx peaks, with the PWMs for the more posterior Hox genes showing highest enrichments.

We then examined the effect of adding the Exd and Hth cofactors on Hox PWM enrichments in the Ubx peaks. The presence of these cofactors results in a dramatic increase in the enrichments for Exd and Hth PWMs (Fig. 4d), and the relative enrichments of the Hox PWMs appear to be shifted towards a greater preference for the Abd-B PWM. Overall this indicates that, in conjunction with Exd and Hth, Ubx can bind at a novel set of sites that have Hox, Exd and Hth motifs and that association with cofactors alters Ubx binding preference.

### Analysis of Hox DNA-binding “fingerprints”

**Fig. 5:**
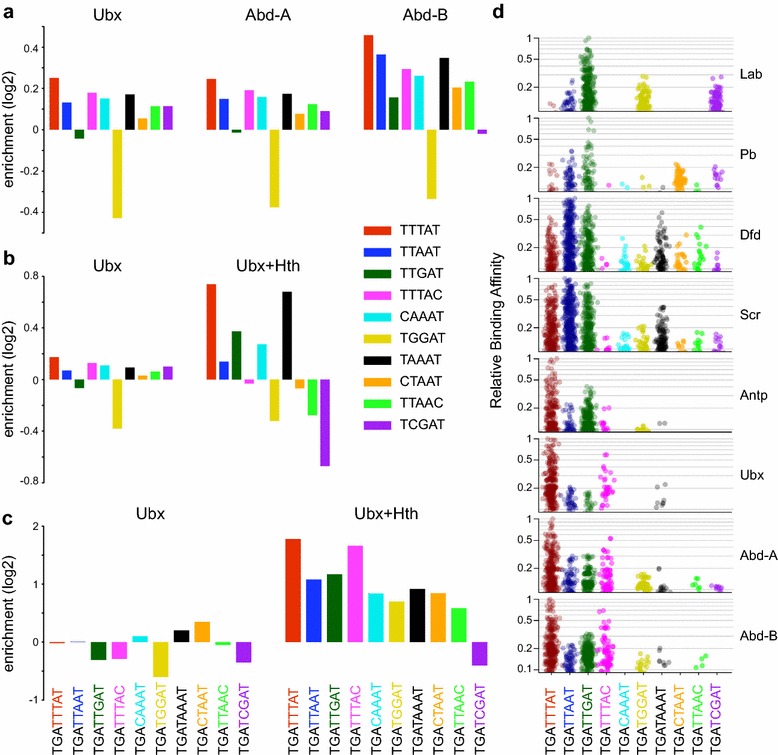
Preferential Hox DNA-binding fingerprints. An enrichment score based on k-mer frequency per kb, in selected peak sets versus background sequence, is plotted for 5-mer and 8-mer sequences derived from the Slattery et al. in vitro SELEX-Seq study on Hox protein binding [9]. **a** Enrichment of a set of Hox monomer binding 5-mers for Ubx (all q-value 1e–2 peaks, Experiment 1), Abd-A (all q-value 1e–2 peaks) and Abd-B (all q-value 1e–2 peaks). Although the Abd-B peaks show higher enrichments, overall the three Hox proteins have very similar 5-mer enrichment profiles. **b** Comparative 5-mer enrichment analysis between Ubx (all q-value 1e–2 peaks, Experiment 2) and Ubx + Hth (328 peaks specific to Ubx + Hth, using q-value 1e–2 peaks) reveals specific changes in the enrichment profile; in the Ubx + Hth peaks, the TTGAT (*dark green*) 5-mer containing the core Exd motif ‘TGAT’ [13] is preferentially enriched, and the TTTAT (*red*) 5-mer containing the core Hox motif ‘TTAT’ is more enriched relative to the TTAAT (*dark blue*) 5-mer containing the core ‘TAAT’ motif. **c** Enrichment of a set of Exd-Hox dimer binding 8-mers for Ubx (all q-value 1e–2 peaks, Experiment 2) and Ubx + Hth (highest 1000 peaks specific to Ubx + Hth from the overlap between Ubx + Hth and Ubx alone, using q-value 1e–2 peaks, Experiment 2). The 8-mer fingerprint of Ubx in the presence of Exd and Hth strongly resembles the posterior Hox class fingerprints from the SELEX-Seq study (see Fig. 5d). The TGATTTAT (*red*) and TGATTTAC (*magenta*) 8-mers clearly relate the in vivo fingerprint to the in vitro posterior Hox fingerprints. **d** Strip charts showing the distribution of relative binding affinities for each of the eight Exd-Hox dimers to a set of core Exd-Hox binding 8-mers. Image was reproduced from the SELEX-Seq study [9], with permission from Elsevier

To further examine the sequence specificity of Hox binding, we measured the enrichment of specific k-mer sequences derived from in vitro SELEX-Seq studies; a set of core Hox binding 5-mers and a set of 8-mer sequences associated with Hox-cofactor binding that have been used to generate sequence preference “fingerprints” for different Hox proteins [9]. Examining the set of Hox binding 5-mers, we find that Ubx, Abd-A and Abd-B show a similar profile, with TTTAT (red) being the most highly enriched and TGGAT (yellow) being depleted relative to background sequences. Abd-B shows higher enrichments and a profile of enrichment that differs slightly from that of Ubx and Abd-A, but overall it appears that in the absence of Exd and Hth, these three Hox proteins bind very similar sequences in vivo (Fig. 5a). For Ubx in the presence of Exd and Hth, the 5-mer enrichment profile is considerably altered compared to Ubx on its own, showing both higher scores and changes in the relative enrichments of particular motifs (Fig. 5b). Some of these changes may be related to the inclusion of Exd and Hth binding motifs in the peak sequences; notably the dark green k-mer containing the core Exd motif TGAT [13] is more enriched. In addition, the red k-mer, containing the core Hox motif TTAT, is relatively more enriched compared to the dark blue k-mer containing the core TAAT motif. This fits with the enhanced enrichment of Abd-B family PWMs (with the Hox core consensus TTAT; [13]) over Ubx family PWMs (with the Hox core consensus TAAT; [13]) noted in the PWMEnrich analysis in Fig. 4d. Overall, this supports a cofactor-dependent modification of Hox binding specificity in vivo that is in line with the “latent specificity” model derived from in vitro data [9].

The SELEX-Seq analysis investigated in vitro Hox binding preferences in the presence of cofactors using full-length Exd and a truncated Hth that contains the Exd interaction domain but not the homeodomain [9]. Based on a set of 8-mer sequences, this analysis indicated that different Hox proteins, in association with Exd and Hth, exhibit preferential binding profiles or “fingerprints” which fall into three classes: an anterior class of Lab and Pb, a central class of Dfd and Scr and a posterior class of Antp, Ubx, Abd-A and Abd-B, each class displaying preferential binding to a distinct combination of 8-mers. We compared the in vivo 8-mer enrichment profile for Ubx binding in the presence of Exd and Hth with the in vitro SELEX-Seq fingerprints, and found that it strongly resembles the posterior class fingerprint (Fig. 5c, d); with TGATTTAT (red) being most enriched, followed by TGATTTAC (magenta), then TGATTGAT (dark green) and TGATTAAT (dark blue). Two additional features are worth mentioning: first, the in vivo Ubx profile resembles the general posterior class fingerprint rather than the Ubx fingerprint specifically (i.e. it does not show the prevalence of TGATAAAT (black) over TGATGGAT (yellow) that is a feature of the in vitro Ubx fingerprint). Second, two 8-mers, TGACAAAT (light blue) and TGACTAAT (orange), are prominent in the in vivo profile but not in the in vitro fingerprint. This is interesting since both these sequences contain TGAC, a core Hth binding motif. The in vitro fingerprints were derived from complexes lacking the Hth DNA-binding domain; the enrichment of the TGAC-containing 8-mers suggests that the specific DNA binding of Hth plays a role in the in vivo targeting of the Ubx-cofactor complex.

### Hox competition with chromatin

**Fig. 6:**
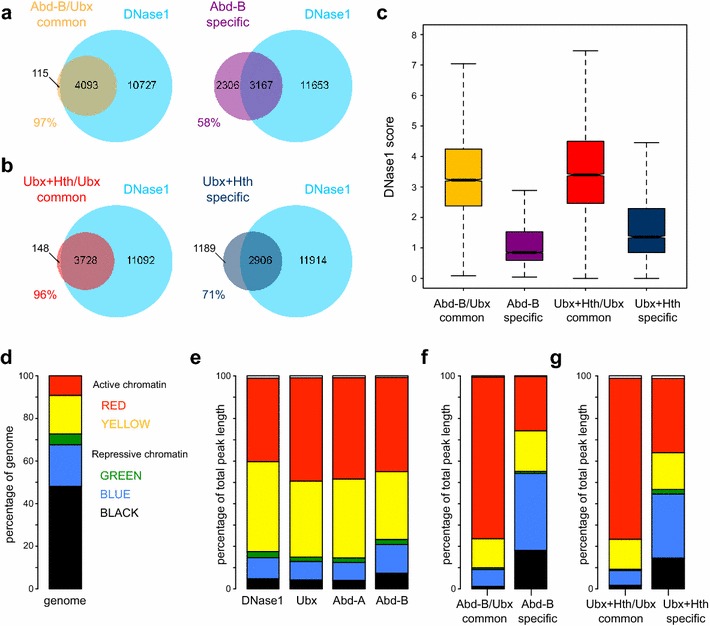
Abd-B specific and cofactor-dependent Ubx peaks in DNase1-inaccessible chromatin. **a** Comparative overlap analysis between Abd-B/Ubx common and Abd-B specific peaks with DNase1 peaks. Almost all (97 %) of the Abd-B/Ubx common peaks overlap with DNase1, while only 58 % of the Abd-B specific peaks overlap with DNase1. Hox peak sets were derived by the overlap analysis of Abd-B and Ubx Experiment 1; using q-value 1e−10 peaks. DNase1 peaks are q-value 1e–2. **b** Comparative overlap analysis between Ubx + Hth/Ubx common and Ubx + Hth specific peaks with DNase1 peaks. Almost all (96 %) of the Ubx + Hth/Ubx common peaks overlap with DNase1, while only 71 % of the Ubx + Hth specific peaks overlap with DNase1. Hox peak sets were derived by the overlap analysis of Ubx + Hth and Ubx Experiment 2; using q-value 1e−10 peaks. DNase1 peaks are q-value 1e–2. **c** For each selected peak set, the median bedGraph score per peak was calculated and the distribution plotted, showing that Abd-B specific and cofactor-dependent Ubx (Ubx + Hth specific) peaks have markedly reduced DNase1 accessibility. Hox peak sets were derived by overlap analysis as described in **a** and **b** but using q-value 1e–2 peaks. **d** Using the *colour-coded* chromatin state classification scheme described in Kc cells [25], the plot shows the prevalence of different chromatin states across the Kc cell genome. **e** Chromatin state prevalence plots for DNase1 (all q-value 1e–2 peaks), Ubx (all q-value 1e–2 peaks, Experiment 1), Abd-A (all q-value 1e–2 peaks) and Abd-B (all q-value 1e–2 peaks). **f** Chromatin state prevalence plots for Abd-B/Ubx common (highest 1000 peaks) and Abd-B specific (highest 1000 peaks). Peak sets were derived as described in **c**. The Abd-B specific peaks are strongly shifted towards the ‘repressed’ *blue* and *black* states. **g** Chromatin state prevalence plots for cofactor-independent Ubx peaks (Ubx + Hth/Ubx common; highest 1000 peaks) and cofactor-dependent Ubx peaks (Ubx + Hth specific; highest 1000 peaks). Peak sets were derived as described in **c**. The cofactor-dependent Ubx peaks are strongly shifted towards the ‘repressed’ states

Whereas the Ubx and Abd-A binding peaks almost completely overlap DNase1 accessible regions, the overlap analysis indicated that a considerable proportion of the Abd-B peaks are not associated with accessible chromatin (Fig. 1b). Binding of Abd-B in DNase1 inaccessible chromatin is particularly associated with the Abd-B specific peaks; while 97 % of the Abd-B and Ubx common peaks overlap DNase1 peaks, only 58 % of the Abd-B specific peaks do (Fig. 6a).

The ability of Abd-B to bind DNase1 inaccessible regions is supported by an analysis of the DNase1-Seq counts in the Hox binding peaks. The Abd-B peaks in common with Ubx show high DNase1 accessibility, whereas the peaks specifically bound by Abd-B show strongly reduced accessibility (Fig. 6c). A similar situation holds for the comparison of Ubx peaks in the absence and presence of Exd and Hth. In the absence of these cofactors, the Ubx peaks largely correspond to DNase1 accessible regions by overlap analysis (Fig. 1b). The common peaks, bound by Ubx both in the absence and presence of the cofactors, show almost complete overlap (96 %) with DNase1 peaks (Fig. 6b) and have high DNase1 accessibility when assessed by DNase1-Seq count analysis (Fig. 6c). In contrast, the Ubx peaks that are cofactor-dependent show less overlap (71 %) with DNase1 peaks (Fig. 6b) and have markedly reduced accessibility when assessed by DNase1-Seq count analysis (Fig. 6c).

This relationship between Hox binding and chromatin accessibility is supported by analysis of the chromatin states associated with bound regions. We used the five-state classification derived from chromatin protein analysis in Kc167 cells [25]. As expected, in comparison to the overall prevalence of the different states across the genome, the DNase1 peaks are preferentially in the “active” red and yellow states (Fig. 6d, e). The chromatin state profile of the Ubx and Abd-A peaks closely resembles that of the DNase1 peaks, whereas for Abd-B the chromatin state profile is shifted towards the repressed blue and black states (Fig. 6e). The Abd-B peaks can be subdivided into those that overlap with the Ubx/Abd-A peaks and those that are Abd-B specific. The overlapping peaks show a clear “active” chromatin profile, similar to DNase1, whereas the Abd-B specific peaks show a profile strongly shifted towards the “repressed” blue and black states (Fig. 6f). A similar situation holds for the comparison of cofactor-independent versus cofactor-dependent Ubx peaks. The cofactor-independent peaks (i.e. the peaks that are common between Ubx binding on its own and Ubx binding in the presence of Exd/Hth) show an “active” chromatin profile, whereas the cofactor-dependent peaks (i.e. the peaks occurring only in the presence of Exd/Hth) show a chromatin profile markedly shifted towards the “repressed states” (Fig. 6g).

**Fig. 7:**
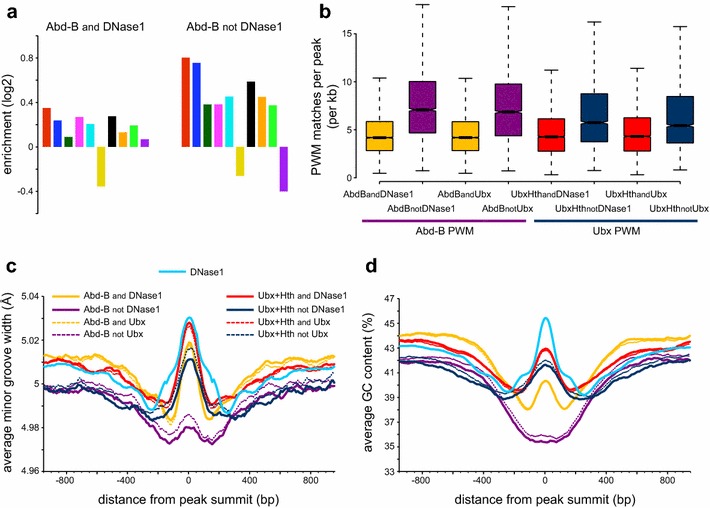
Characteristics of Abd-B peaks in DNase1-inaccessible chromatin. **a** Enrichment of a set of core Hox binding 5-mers (as described in Fig. 5) in Abd-B peaks in accessible (Abd-B and DNase1) and inaccessible (Abd-B not DNase1) chromatin. Peak sets were derived by the overlap analysis of Abd-B and DNase1; using q-value 1e–2 peaks. Although the DNase1-inaccessible peaks generally have higher 5-mer enrichments, the profiles are similar. **b** Density analysis of matches to the Abd-B PWM (MA0165.1, JASPAR database) on the left and to the Ubx PWM (MA0094.2, JASPAR database) on the *right*. The Abd-B peaks in inaccessible chromatin (AbdBnotDNase1) and the Abd-B specific peaks (AbdBnotUbx) have a higher density of matches to the Abd-B PWM compared to peaks in accessible chromatin (AbdBandDNase1) or Abd-B/Ubx common peaks (AbdBandUbx). Similarly, the cofactor-dependent Ubx peaks (UbxHthnotUbx) have a higher density of matches to the Ubx PWM compared to cofactor-independent peaks (UbxHthandUbx). Peak sets were derived by the overlap analysis of Abd-B and DNase1, Abd-B and Ubx Experiment 1, Ubx + Hth and DNase1, and Ubx + Hth and Ubx Experiment 2; using q-value 1e–2 peaks. **c** Comparative DNA shape analysis, showing that the average predicted minor groove width for Abd-B peaks in inaccessible chromatin (Abd-B not DNase1) is narrower in a region of 200 bp centred on the peak summit, compared to the Abd-B peaks in accessible chromatin (Abd-B and DNase1) where the average predicted minor groove width is wider. This narrowing in the minor groove width is not observed in the Ubx + Hth peaks occurring in inaccessible chromatin. DNase1 peak summits have a local increase in minor groove width. Peak sets were derived as described in **b**. DNase1 peaks are q-value 1e−2. **d** Comparative GC composition analysis, showing that Abd-B peaks in inaccessible chromatin (Abd-B not DNase1) are associated with a lower GC content compared to Abd-B peaks in accessible chromatin (Abd-B and DNase1). This lower GC content is not observed in the Ubx + Hth peaks occurring in inaccessible chromatin. DNase1 peak summits have a prominent local increase in GC content. Peak sets are the same as in **c**

Overall, these data indicate that, although much of the Hox binding is to open chromatin, there are two situations where Hox proteins can bind in less-accessible regions: Abd-B specific binding and cofactor-dependent Ubx binding. In the latter situation, the interaction between Ubx and the cofactors Exd/Hth provides a rationale for the ability of Ubx to bind in less-accessible chromatin. However what is the basis for Abd-B’s specific ability to access a set of relatively closed chromatin regions? One possibility is that Abd-B can uniquely recruit a cofactor present in Kc167 cells; while we do not have strong evidence against the Abd-B cofactor idea, we do not see a clear candidate cofactor binding motif co-enriched with Hox motifs in these regions. Alternatively these regions may contain high affinity binding sites uniquely for Abd-B. We looked at 5-mer enrichments, comparing Abd-B peaks in accessible chromatin (where Abd-B largely binds in common with Ubx and Abd-A) with Abd-B peaks in DNase1-inaccessible regions and find that, although 5-mer enrichments are generally higher for the latter, the relative enrichments of the different k-mers are similar (Fig. 7a).

We identified two potentially relevant features of the Abd-B specific peaks associated with closed chromatin. First, as compared to the peaks in accessible chromatin, they have a higher density of matches to the Abd-B PWM (median 6.5 matches/kb for the “Abd-B not DNase1” peaks versus 4.5 matches/kb for the “Abd-B and DNase1” peaks, p = 0.001; Fig. 7b). Second, we find that the DNA shape profile differs between the Abd-B peaks in accessible versus inaccessible chromatin. DNA shape, particularly the width of the minor groove, has been proposed to play a major role in the specificity of Hox binding as the N-terminal arm of the homeodomain makes specific electrostatic contacts within the minor groove [22, 23]. We find that the Abd-B peaks in accessible chromatin have a predicted wider minor groove in a region of about 200 bp centred on the peak summits. As these peaks are associated with chromatin accessibility, we investigated the DNase1 peaks themselves and find that this local increase in minor groove width is a general feature of DNase1 accessible regions. In contrast, the Abd-B peaks in closed chromatin have a narrower minor groove (Fig. 7c). A narrow minor groove has been associated with Hox binding affinity and specificity as it allows for stronger ionic interactions [26, 27]. This difference in DNA shape is also associated with a lower GC content for the Abd-B peaks in closed chromatin (Fig. 7d).

## Discussion

We have performed a comparative analysis of Hox protein binding specificity in the context of chromatin in *Drosophila* tissue culture cells, extending previous in vitro analyses of Hox protein specificity and revealing a strong influence of chromatin accessibility on Hox binding. Our investigation also allowed an in vivo assessment of previously defined in vitro Hox binding preferences and an analysis of the role of the major Hox cofactors, Exd and Hth.

While it has been accepted for some time that the nucleosomal organization of chromatin is likely to play a major role in restricting transcription factor binding in the genome (reviewed in [28]), the issue has been brought into focus by the desire to predict transcription factor binding on the basis of sequence, and the realization from genome-wide ChIP studies, that generally only a very small fraction of predicted transcription factor binding sites are actually occupied in vivo. Strong association between chromatin accessibility and the binding of a variety of transcription factors has been observed [17] although it is difficult to separate cause and effect in studies on developmental tissues. In experiments on inducible transcription factor binding, the prior chromatin accessibility state has been clearly shown to dictate the binding sites for Heat Shock Factor and the glucocorticoid receptor [29, 30]. In contrast, the reprogramming factors Oct4, Sox2 and Klf4 preferentially bind closed chromatin [31]. In our studies, using exogenous expression of Hox proteins in a cell line, we also relate the Hox binding profiles to the pre-existing state of chromatin accessibility. We find that Ubx and Abd-A binding is almost exclusively in accessible chromatin, whereas Abd-B can additionally bind to regions whose accessibility is low prior to the expression of Abd-B. Interestingly, Ubx is also able to bind in less-accessible chromatin in the presence of the Exd and Hth cofactors. These results confirm expectations that nucleosomes play a major role in restricting Hox protein access to binding sites [14, 32], but also reveal circumstances where Hox proteins can effectively compete with nucleosomes to access sites residing in DNase1 inaccessible chromatin. This indicates that a key issue in understanding Hox binding specificity is how Hox proteins compete with nucleosomes and this is a major question for the understanding of transcription factor binding in general. Our observation of two different situations where Hox proteins can effectively compete provides insight into this process. We have investigated the sequence characteristics of the subset of Abd-B peaks occurring in inaccessible chromatin to probe the basis of Abd-B’s pioneer activity at these sites. One possibility is that these sites contain particularly high affinity Abd-B DNA-binding motifs; however at least when using a set of Hox monomer binding 5-mers we find little evidence for this, since the enrichment profile of these 5-mers is very similar for Abd-B peaks occurring in accessible and inaccessible chromatin. We did however find an increased density of matches to the Abd-B PWM in peaks occurring in inaccessible chromatin, suggesting that a critical factor may be the number of Abd-B molecules able to bind in a particular region. We also observed an interesting connection to DNA shape. We show that DNase1 accessible regions are generally associated with a small local increase in DNA minor groove width over a region of about 200 bp centred on the DNase1 peak summits (Fig. 7c). This is potentially important as wider minor grooves decrease the negative electrostatic potential of the DNA and are associated with weaker histone-DNA interactions [27, 33]. While the Abd-B/Ubx common peaks also show a similar local widening of minor groove width, the Abd-B specific sites do not. The Abd-B specific sites tend to have narrower minor grooves, a feature that is associated with increased Hox protein binding affinity due to enhanced electrostatic interaction with the N-terminal arm of the homeodomain [26, 34]. The relative effect of DNA shape on nucleosome stability versus homeodomain binding affinity may set in place a fine competitive balance for binding at these sites. For Ubx, the provision of the Hox cofactors Exd and Hth allows Ubx to bind at sites in DNase1 inaccessible chromatin that are not bound in the absence of the cofactors. This provides strong in vivo evidence supporting the key role these cofactors play in Hox protein binding as suggested by in vitro studies [3–5]. It also suggests that an important role of cofactors in vivo involves the establishment of Hox binding through competition with nucleosomes at target sites. The cofactor-dependent Ubx peaks are associated with enrichment of Hox, Exd and Hth binding motifs indicating the involvement of the binding of several proteins. The common theme we see between the two types of binding in DNase1 inaccessible chromatin, Abd-B specific peaks and cofactor-dependent Ubx peaks, is the multiple binding of proteins at these sites. The combined effect of multiple protein binding events may be the basis for effective competition with nucleosomes, consistent with the collaborative competition mechanism proposed by Miller and Widom [35].

One caveat to our interpretation of the Abd-B specific binding and cofactor-dependent Ubx binding in DNase1 inaccessible chromatin is the possibility of indirect effects. For example, Abd-B could activate the expression of a transcription factor responsible for opening the chromatin at novel sites. Although it is difficult to exclude indirect effects, we see no evidence for them. We do not find a clear novel DNA motif at the Abd-B specific sites. Also, the cofactor-dependent Ubx peaks show strong enrichment of Exd and Hth motifs, consistent with a direct effect.

Extensive in vitro analysis of Hox DNA-binding preferences has identified that, while they all bind short AT-rich sequences, the individual Hox proteins have subtly different preferred motifs [12, 13]. We find some support for the relevance of these preferences for in vivo binding in that the binding peaks of Ubx, Abd-A and Abd-B all show better enrichment scores for PWMs of posterior Hox proteins (Ubx, Abd-A and Abd-B) compared to PWMs of anterior Hox proteins (Lab, Dfd and Scr). However, within the posterior class there is no clear correlation between the in vitro preferences and the in vivo enrichments since the Abd-B PWMs show the highest enrichment for all three of the posterior Hox proteins we assayed. Overall, we do not see clear evidence for distinct motifs bound by Ubx, Abd-A or Abd-B.

In vitro, DNA-binding specificity of Hox proteins is enhanced by the Exd and Hth cofactors [3–5], and we identify a Hox/Exd consensus binding motif (5′-TGATTTAT-3′) as the most enriched motif in de novo motif searches on the cofactor-dependent Ubx peak sequences (Additional file 1: Figure S3). The cofactor-dependent Ubx binding peaks show an 8-mer enrichment fingerprint that closely matches the in vitro SELEX-Seq Ubx-Exd fingerprint [9], with clear features that place it in the posterior Hox class. However, the match is to the posterior Hox class as a whole rather than specifically to the Ubx fingerprint.

In terms of in vivo target specificity, we find that Ubx and Abd-A bind at very similar sites, whereas Abd-B binds, in addition, at a specific set of targets. This fits well with the in vivo functions of these Hox proteins. Ectopic expression of Ubx and Abd-A in embryos specifies similar anterior abdominal segments, whereas Abd-B expression determines the more distinct posterior abdomen [36–39]. In wing imaginal discs, ectopic expression of either Ubx or Abd-A generates very similar transformations of wing into haltere, whereas Abd-B generates only a partial transformation into haltere-like tissue [40]. Abd-B is also at the top of the posterior dominance hierarchy and its effects can over-ride those of Ubx and Abd-A [41, 42].

A close connection between chromatin accessibility and Hox function is also indicated by experiments comparing chromatin accessibility in Hox-regulated tissues. For example, the chromatin accessibility profile of wing and haltere imaginal discs are strikingly similar [43]. Thus although Ubx specifies haltere rather than wing development, this regulation of appendage morphology occurs with little change to the genomic open chromatin profile, suggesting that Ubx acts predominantly on regulatory elements that are already accessible in the “ground state” wing developmental program.

Our experiments focus on Hox binding rather than overall Hox function and we demonstrate that Hox specificity is represented at the level of binding with the differential binding of Ubx/Abd-A versus Abd-B. The focus on binding also allows us to show that Hox cofactors do not only affect Hox function but also clearly affect the target specificity of Hox proteins in vivo, enabling Ubx to bind cofactor-dependent targets that it cannot bind on its own.

## Conclusions

We have established a flexible platform for the analysis of target specificity of Hox transcription factors in the *Drosophila* Kc167 cell line. This enables us to move beyond in vitro studies of Hox binding specificity to perform genome-wide binding analysis on an in vivo chromatin substrate. In this system, we show that Hox proteins exhibit specific binding in the absence of the canonical Hox cofactors Exd and Hth, with Abd-B targeting sites that are not bound by either Ubx or Abd-A. Nevertheless, Exd and Hth can have a strong impact on target specificity and their provision enables Ubx to bind at a novel set of targets that it cannot access in their absence. In contrast to the common Hox binding sites, both the Abd-B specific sites and the cofactor-dependent Ubx sites are in relatively closed chromatin indicating that competition with nucleosomes may play a key role in determining Hox target specificity. The involvement of chromatin in Hox binding has implications for the specificity of the downstream gene expression response to Hox regulation. This may be particularly important for the regulation of different sets of target genes in specific tissue types or particular tumour cells where much of the target gene selection may be based on differences in the chromatin landscape in the different cells.

## Methods

### Cell culture

Kc167 cells (obtained from the Drosophila Genomics Resource Center) were cultured in Schneider’s medium supplemented with 5 % foetal calf serum and antibiotics at 25 °C.

### Expression plasmid cloning

The Hox cDNA clones were obtained from the Berkeley Drosophila Genome Project (BDGP) Gold collection provided by the Drosophila Genomics Resource Center: Ubx (clone RE43738 encoding protein isoform E), Abd-A (clone RE04174 encoding protein isoform common to transcripts A/C/D), Abd-B (clone RE47096 encoding protein isoform common to transcripts A/C/D/E: the Abd-B clone had a single point deletion of an A at position 1289 (position 578 of the CDS), which was reintroduced by site-directed mutagenesis). To generate the GFP-tagged Hox expression vectors, the eGFP CDS (with stop codon removed) was cloned upstream of each Hox CDS and the resulting fusion cloned as an EcoRI-XbaI fragment in the MCS of the pMTA expression vector (Invitrogen V4120-20) under the control of the inducible *Drosophila* metallothionein promoter. The Hth cDNA clone (protein isoform A) was obtained from Richard Mann. For the co-expression of Ubx and Hth, we used a bicistronic expression vector employing the 2A peptide self-cleavage system. The Hth CDS (with stop codon removed) was cloned upstream of the eGFP-Ubx CDS, separated by the T2A peptide sequence used in pAc5-STABLE2-Neo [44]. The resulting fusion was cloned as an EcoRI-ApaI fragment in the MCS of the pMTA expression vector. To generate Ubx with mutations affecting DNA-binding, the following mutations were introduced in the Ubx homeodomain sequence: R3A, R5A, I47A, Q50K and N51A. For more detailed information about the Hox constructs used in this study see Additional file 2.

### Transfection, induction of gene expression and fixation

Cells harvested in log phase were used to seed two 10 cm dishes per sample (Nunclon™ Delta treated, Nunc 150350) at a density of 2 × 10^7^ cells per dish. We performed two experiments; Ubx, Abd-A and Abd-B samples were seeded using the same harvested cells in Experiment 1 and Ubx, mutant Ubx and Ubx + Hth likewise in Experiment 2. Cells were allowed to settle and adhere to the dish surface for about 3 h, and transfection was performed using FuGENE^®^ 6 Transfection Reagent (Promega E2691) according to the manufacturer’s instructions. For each dish, 1.5 ml of 1.5:1 FuGENE^®^: DNA mix was prepared in a 5 ml polypropylene tube (BD Falcon 352063) by adding 45 µl FuGENE^®^ and 30 µg DNA (150 µl of 0.2 µg µl^−1^ DNA prepared in TE buffer) to 1305 µl Opti-MEM^®^ I reduced-serum medium (Invitrogen 11058-021). The FuGENE^®^ DNA mix was then added to 10 ml Schneider’s medium and mixed thoroughly. For each seeded dish, medium was removed and replaced by the entire volume of Schneider’s medium containing FuGENE^®^ DNA mix (~11.5 ml). Dishes were incubated at 25 °C for ~12 h before inducing gene expression. For induction, medium was removed and replaced by 10 ml of 500–1000 µM CuSO_4_ in Schneider’s medium. Dishes were incubated at 25 °C for 4 h. Cells were harvested, spun down at 300*g* for 3 min, and fixed using 1 % formaldehyde (Sigma-Aldrich F8775) in PBS for 10 min at 23 °C in an Eppendorf Thermomixer at 700 rpm. Fixation was stopped by spinning at 3000*g* for 1 min, placing the samples immediately on ice, washing once in PBS/125 mM Glycine/0.01 % Triton X-100 and twice in PBS/0.01 % Triton X-100.

### FACS

Samples were filtered (30 µm, Partec 04-004-2326) and diluted to an appropriate concentration for FACS sorting by adding PBS/0.01 % Triton X-100 to a final volume of 3 ml. Cells were sorted using a 70 µm nozzle on a MoFlo FACS machine (Beckman Coulter) equipped with a 488 nm argon laser (100 mW). Cells were sorted into PBS/0.01 % Triton X-100. An equal number of cells (~10^6^) were sorted for all samples. Events were triggered on forward scatter and GFP+ events were sorted using the gating strategy described in Additional file 1: Figure S1. Data were acquired and analysed using Summit software (Beckman Coulter).

### Estimation of Hox protein expression level

Protein expression level was estimated for Ubx-GFP by FACS using AcGFP Flow Cytometer Calibration Beads (Clontech 632594). The excitation/emission spectra and brightness for AcGFP and the eGFP used in the Hox-GFP constructs are almost identical. Cells were transfected, induced and fixed as for ChIP-Seq then analysed by FACS using the same gating strategy as the ChIP-Seq samples. This was used to derive the percentiles of the fluorescence intensity profile corresponding to the sorted cells. Cells from the same transfected and induced culture were also analysed unfixed allowing comparison to the calibration beads. The bead intensities were used to generate a calibration curve of number of GFP molecules (Molecular Equivalent of Soluble Fluorophore; MESF) versus fluorescence intensity, from which the number of GFP molecules per cell was estimated for the sorted GFP+ cells at the appropriate percentiles (subtracting the number of molecules estimated for the GFP− background).

### ChIP on sorted cells

Sorted cells were pelleted in a swing-out rotor at 4000*g* for 15 min at 4 °C, transferred to a microfuge tube, pelleted again and re-suspended in 100 µl Lysis Buffer (50 mM Tris.HCl (pH 8), 10 mM EDTA.Na_2_, 1 % SDS) containing protease inhibitors (Sigma-Aldrich P8340) and sonicated for 9 cycles at high setting using a Diagenode Bioruptor (1 cycle is 30 s ON and 30 s OFF). For chromatin preclearing and the ChIP reaction, Protein A-Sepharose beads (adjusted to a 50 % (v/v) concentration in 20 % ethanol, Sigma-Aldrich P9424) were treated as follows: washed in 1 ml Buffer A (10 mM Tris.HCl (pH 7.5), 1 mM EDTA.Na_2_, 140 mM NaCl, 1 % Triton X-100, 0.1 % SDS, 0.1 % Na-deoxycholate), preblocked for 2 h while mixing in 0.75 mg ml^−1^ BSA in 1 ml Buffer A, spun down and re-suspended in Buffer A to give a final bead concentration of 50 % (for preclearing) or 12.5 % (for the ChIP reaction). Chromatin was precleared by adding 25 µl of 50 % preblocked beads to each 100 µl chromatin sample and incubating for 15 min at 4 °C in an Eppendorf Thermomixer at 1400 rpm. The beads were then pelleted and 100 µl of the precleared chromatin supernatant transferred to a fresh microfuge tube. An equal volume of the chromatin supernatant (~1 µl) was retained from each of the Hox samples and combined to represent the input, which was purified alongside the ChIP samples. To each 100 µl precleared chromatin sample, 200 µl RIPA buffer (16.7 mM Tris.HCl (pH 8), 1.2 mM EDTA.Na_2_, 167 mM NaCl, 1.1 % Triton X-100, 0.01 % SDS) containing protease inhibitors was added, the resulting solution mixed thoroughly, and 1 µl of 0.1 mg ml^−1^ affinity-purified rabbit anti-GFP antibody [45] added. Samples were incubated overnight while mixing at 4 °C. The ChIP reaction was performed by adding 100 µl of 12.5 % preblocked beads to each 300 µl chromatin sample and incubating for 40 min at 4 °C in an Eppendorf Thermomixer at 1400 rpm (30 s ON and 3 s OFF). The beads were pelleted, rinsed in 1 ml Buffer A, then sequentially washed in 1 ml of each of the following wash buffers for 5 min at 4 °C while mixing: once in Buffer B (20 mM Tris.HCl (pH 8), 2 mM EDTA.Na_2_, 150 mM NaCl, 1 % Triton X-100, 0.1 % SDS), four times in Buffer C (20 mM Tris.HCl (pH 8), 2 mM EDTA.Na_2_, 500 mM NaCl, 1 % Triton X-100, 0.1 % SDS), once in Buffer D (10 mM Tris.HCl (pH 8), 1 mM EDTA.Na_2_, 250 mM LiCl, 1 % NP40, 1 % Na-deoxycholate), and twice in Buffer E (10 mM Tris.HCl (pH 8), 1 mM EDTA.Na_2_). The beads were then re-suspended in 150 µl Elution Buffer (50 mM NaHCO_3_, 1 % SDS), vortexed for 15 min at room temperature and pelleted. A volume of 145 µl of the supernatant containing the eluted chromatin was collected in a fresh microfuge tube then another 150 µl Elution Buffer was added to the beads and the process repeated. The combined volume of supernatant collected was made up to a total volume of 300 µl with Elution Buffer. To the input chromatin, Elution Buffer was added to make up a total volume of 300 µl. RNase A (1 µl for the ChIP samples, 2 µl for the input sample) and 24.3 µl of 4 M NaCl were added to each sample. Samples were incubated at 67 °C for 3 h, Proteinase K (20 mg ml^−1^, 2 µl for the ChIP samples, 4 µl for the input sample) was added and samples were incubated for another 2 h at 67 °C. The volume of each sample was made up to 500 µl by adding 50 mM Tris.HCl (pH 8), 10 mM EDTA.Na_2_. DNA was purified by phenol–chloroform extraction and ethanol precipitation using linear acrylamide as carrier and re-suspended in 10 mM Tris.HCl pH 8.5 (10 µl for the ChIP samples, 30 µl for the input sample).

### Sequencing of ChIP DNA

Samples were processed and sequenced by Source BioScience. The Illumina TruSeq ChIP Sample Preparation Kit was used to generate indexed paired-end sequencing libraries in accordance with the manufacturer’s guide (Rev. A, August 2012) except that no size selection was performed. For the ChIP samples, the entire 10 µl volume of ChIP DNA was used for library preparation and 17 cycles of amplification were performed. For the input sample, 5-10 ng of input DNA was used for library preparation and 12 cycles of amplification were performed. Samples were either sequenced on the Illumina MiSeq (Experiment 1; Ubx, Abd-A, Abd-B and input) or HiSeq 2000 (Experiment 2; Ubx, mutant Ubx and Ubx + Hth) platforms to generate 36 bp or 100 bp reads, respectively.

### ChIP-Seq data processing

We performed two biological replicates for each sample, except in the case of Ubx where we performed two replicates for Experiment 1 and one replicate for Experiment 2. For all samples, Experiment 1 input chromatin was used as the reference control to assay ChIP enrichment. All analyses are based on the *Drosophila melanogaster* genome BDGP release 5. Sequencing reads in the raw fastq files were mapped using Bowtie1 [46] using default paired-end settings, a maximum insert size of 1000 bp for valid paired-end alignments (-X 1000) and reporting only uniquely mapped reads (-m 1). Reads in the output SAM files were processed using Samtools [47] to generate BAM files. Reads from the individual sample replicates were then combined, and filtered using Bedtools [48] to remove the over-represented reads found at the exons of Ubx, Abd-A, Abd-B and Hth; artefacts of the expression vectors used in the transfections. MACS2 [49] was used for peak calling using default settings, the input sample specified as the control file (-c), the -f parameter set to process paired-end BAM files (-f BAMPE), a band width of 200 bp (--bw) and a q-value of 1e−2 or 1e−10 (-q). Binding profiles were generated as fragment pileup tracks in bedGraph format with pileup signal normalized per million reads (-B -SPMR). Only peaks in euchromatin (chr2L, chr2R, chr3L, chr3R, chr4, chrX and chrM) were used for downstream analyses. The Kc DNase1 reads [24] were mapped using default single-end settings and MACS2 peak calling performed using default settings without a control file (-f BAM --bw 200 -q 1e−2 -B --SPMR). Binding and chromatin accessibility profiles were visually examined using the Integrated Genome Browser (http://bioviz.org/igb/index.html).

### Overlap and differential peak analysis

Overlap peak analysis was performed using Bedtools [48] intersect; common peaks were defined as having a minimum of 5 % reciprocal overlap (-f 0.05 -r -u) and specific non-overlapping peaks were reported using the -v option (-f 0.05 -r -v). Overlap analysis was used to generate the common and specific peak sets used in Fig. 5c (for Ubx + Hth), Fig. 6a–c, f, g, and Fig. 7a–d. Overlap analysis was also used in Fig. 6e–g to determine, per given peak set, the percentage of total peak length overlapping each of the five chromatin colour states described by Filion et al. [25]; unknowns (shown in white) represent peaks that map to regions in the genome non-annotated with a chromatin state colour. Differential peak analysis was performed using the bdgdiff module in MACS2 [49] to generate the following peak sets: the differential Hox versus DNase1 peak sets in Fig. 4a–c, the common and differential Ubx + Hth versus Ubx peak sets in Fig. 4d, and the differential Ubx + Hth versus Ubx peak set in Fig. 5b (for Ubx + Hth).

### Motif analysis

HOMER [50] and MEME-ChIP [51] were used to perform motif discovery on the cofactor-dependent Ubx peak sequences (Additional file 1: Figure S3). The PWMEnrich R package [52] was used for Hox and cofactor PWM enrichment analysis; motif enrichment scores [log_10_(1/p-value)] were grouped according to transcription factor and plotted as one dimensional dot plots together with median using R. The Biostrings R package [53] was used to count the number of occurrences of selected k-mers derived from the SELEX-Seq study [9] in the sequences underlying given peak sets; the frequency per kb of each k-mer was counted and reported as log_2_(frequency in peak sequences/background frequency) using 2 kb sequences upstream of TSSs from *D. melanogaster* genome BDGP release 5 as background (this is the same background as used for PWMEnrich). FIMO [54] was used for Hox motif density analysis; the number of matches to the Abd-B or Ubx PWMs (MA0165.1 and MA0094.2 from the JASPAR database, respectively) was counted, using a p-value threshold of 0.001, in the sequences underlying given peak sets, and the density is reported as [(number of matches per peak/peak length) × 1000].

### DNA shape analysis

Minor groove widths were predicted using the DNAshape online tool [55] using 2 kb sequences centred on the peak summits. For each base position, the average width was calculated, then the resulting profile was smoothed using a 100 bp sliding window and a step of 10 bp using the ‘rollapply’ function from the zoo R package.

### GC composition analysis

Average GC content was calculated using a Perl script from the Berkeley Drosophila Transcription Network Project (base_composition_across_peaks.pl written by Stewart MacArthur) using 2 kb sequences centred on the peak summits, a window size of 100 bp and a step of 10 bp.

### Immunolabelling

Cells transfected with the pMT-Hth2AGFPUbx bicistronic expression vector were induced for 4 h as described above, and fixed using 4 % formaldehyde (Sigma-Aldrich F8775) in PBS for 10 min at 23 °C in an Eppendorf Thermomixer at 700 rpm. Transfected (GFP+) and non-transfected (GFP−) cells were sorted as described above. Sorted cells were seeded onto 1 × 0.8 cm coverslips in the wells of a 12-well plate each containing 1 ml PBS. Cells were allowed to adhere for 30 min. The PBS was then replaced by 1 ml PTX (PBS/0.5 % Triton X-100) and cells were incubated at room temperature for 30 min. Coverslips were then transferred to microfuge tubes containing mouse anti-Exd B11M monoclonal antibody (1 ml, 1:5 in PTX; [56]) and incubated at 4 °C overnight. Coverslips were then washed three times in PTX then incubated with goat anti-mouse Alexa 568 secondary antibody (1 ml, 1:500, Invitrogen) and 1 µg ml^−1^ DAPI in PTX at room temperature for 2 h. Coverslips were then washed three times in PTX and mounted in Citifluor AF1 Glycerol/PBS solution (Agar Scientific) for immunofluorescence microscopy (Nikon Eclipse TE2000-E confocal).

### Data availability

The ChIP-Seq data are available from GEO under accession number GSE69796. Table S1 in Additional file 1 provides summary statistics of read mapping and data processing.

## Additional files

10.1186/s13072-015-0049-x FACS gating strategy and additional figures
10.1186/s13072-015-0049-x Details of the Hox-GFP constructs

## Abbreviations

TALE: three amino acid loop extension
GFP: green fluorescent protein
ChIP-Seq: chromatin immunoprecipitation with massively parallel sequencing
FACS: fluorescence-activated cell sorter
SELEX-Seq: systematic evolution of ligands by exponential enrichment with massively parallel sequencing
PWM: position weight matrix
bp: base pair
kb: kilobase
MCS: multiple cloning site
SDS: sodium dodecyl sulphate
BSA: bovine serum albumin
rpm: revolutions per minute
