# Supplementary material for: Roles of cofactors and chromatin accessibility in Hox protein target specificity
Source: Epigenetics Chromatin. 2016 Jan 8;9:1. doi: 10.1186/s13072-015-0049-x (PMC4705621; doi:10.1186/s13072-015-0049-x)
Supplement: Supplementary file 1 — 10.1186/s13072-015-0049-x FACS gating strategy and additional figures [file 13072_2015_49_MOESM1_ESM.docx]

**Roles of Cofactors and Chromatin Accessibility in Hox Protein Target Specificity**

**Ching Yew Beh^1^**^†^**, Sherif El-Sharnouby^2^**^†^**, Aikaterini Chatzipli^23^, Steven Russell^34^, Siew Woh Choo^1*^ and Robert White^2*^**

^1^Department of Oral Biology and Biomedical Sciences, Faculty of Dentistry, University of Malaya, 50603 Kuala Lumpur, Malaysia

^2^Department of Physiology, Development and Neuroscience, University of Cambridge, Downing Street, Cambridge, CB2 3DY United Kingdom

^3^Department of Genetics, University of Cambridge, Downing Street, Cambridge, CB2 3EH United Kingdom

^4^Cambridge Systems Biology Centre, University of Cambridge, Tennis Court Road, Cambridge, CB2 1QR United Kingdom

^†^These authors contributed equally to this work.

*Corresponding authors: Siew Woh Choo and Robert White.

Ching Yew Beh [behchingyew@gmail.com](mailto:behchingyew@gmail.com)

Sherif El-Sharnouby [bionouby@hotmail.com](mailto:se266@cam.ac.uk)

Aikaterini Chatzipli [chatzipli@gmail.com](mailto:chatzipli@gmail.com)

Steven Russell [sr120@hermes.cam.ac.uk](mailto:sr120@hermes.cam.ac.uk)

Siew Woh Choo [lchoo@um.edu.my](mailto:lchoo@um.edu.my)

Robert White [rw108@cam.ac.uk](mailto:rw108@cam.ac.uk)

**Additional File 1**

Additional File 1 contains three supplementary figures and one supplementary table.

**Figure S1. FACS gating strategy used to sort transfected Kc cells.** (**A-C**) Gates R1, R2 and R3 were used to sort GFP^+^ Kc cells; A showing all events, B showing events after gating by R1 and C showing events after gating by both R1 and R2. (**D-E**) FACS histograms showing the GFP fluorescence intensity profiles for the different samples of Experiment 1 (D) and Experiment 2 (E). The fluorescence intensity range of the sorted GFP^+^ population is indicated by vertical dashed lines. Note that all samples have a comparable distribution of GFP fluorescence intensity within the sorted range. For the particular replicate of samples shown in D, the sorted GFP^+^ cells constituted 10.8% (Ubx), 9.9% (Abd-A) and 5.4% (Abd-B) of the total events. For the particular replicate of samples shown in E, the sorted GFP^+^ cells constituted 5.8% (Ubx), 6.0% (Ubx mutant) and 4.6% (Ubx+Hth) of the total events. Sort purity > 99%. (**F**) FACS histogram showing the GFP^+^ (in green) and GFP^−^ (in white) populations sorted to assess Hth functionality by Exd labelling (see Figure 3B). The GFP^−^ cells were sorted using gates R1, R2 and R4 (shown in A-C).

**Figure S2. Reproducibility of ChIP assays for Ubx, Abd-A and Abd-B from two independent replicates.** Scatter plots showing Pearson’s correlations between binding profiles for the pairs of Ubx, Abd-A and Abd-B replicates based on binding score per 1 kb window. The two independent ChIP replicates for each of Ubx, Abd-A and Abd-B are highly similar with R > 0.9.


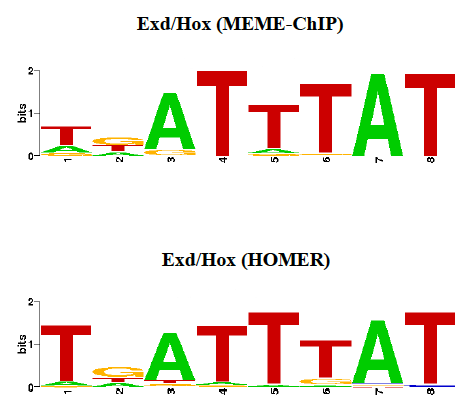


**Figure S3. The discovered Exd/Hox motif in Ubx cofactor-dependent peak sequences.** The Exd/Hox motif was identified using MEME-ChIP and HOMER software. STAMP [57] was used to generate the LOGOs using nucleotide frequency for the *Y*-axis. The number of sequences used to generate each LOGO was 147 (MEME-ChIP) and 170 (HOMER).

**Table S1.** **Summary statistics of read mapping and data processing.** For each dataset, the number of raw reads, uniquely mapped reads, and uniquely mapped reads after removing reads coming from plasmid sequences (clean reads), as well as the number of peaks called at given q-value thresholds, are shown.

|  | Experiment 1 | | | | Experiment 2 | | |  |
| --- | --- | --- | --- | --- | --- | --- | --- | --- |
| Dataset | Ubx | Abd-A | Abd-B | Input | Ubx | Ubx mutant | Ubx+Hth | DNase1 |
| Number of raw reads | 25,478,538 | 23,133,718 | 32,625,412 | 28,074,578 | 37,150,912 | 170,295,836 | 217,660,162 | 28,664,264 |
| Number of uniquely mapped reads | 13,134,060  (51.5%) | 13,844,104  (59.8%) | 21,326,696  (65.4%) | 15,889,222  (56.6%) | 21,201,568  (57.1%) | 88,735,344  (52.1%) | 119,744,776  (55%) | 19,057,945  (66.5%) |
| Number of reads removed due to plasmid exon sequences | 106,751 | 101,183 | 84,557 | 359,796 | 123,695 | 490,391 | 945,875 | - |
| Number of clean reads | 13,027,309 | 13,742,921 | 21,242,139 | 15,529,426 | 21,077,873 | 88,244,953 | 118,798,901 | 19,057,945 |
| Number of peaks at q-value 1e-2 (euchromatin^a^) | 8,604 | 9,029 | 12,322 | - | 8,672 | 9,346 | 11,698 | 14,820 |
| Number of peaks at q-value 1e-10 (euchromatin^a^) | 4,830 | 5,656 | 9,681 | - | 4,218 | 1,793 | 7,971 | - |

^a^Only peaks in euchromatin (chr2L, chr2R, chr3L, chr3R, chr4, chrX and chrM) were retained for downstream analyses.
