# Supplementary material for: Roles of cofactors and chromatin accessibility in Hox protein target specificity
Source: Epigenetics Chromatin. 2016 Jan 8;9:1. doi: 10.1186/s13072-015-0049-x (PMC4705621; doi:10.1186/s13072-015-0049-x)
Supplement: Supplementary file 2 — 10.1186/s13072-015-0049-x Details of the Hox-GFP constructs [file 13072_2015_49_MOESM2_ESM.pdf]

Roles of Cofactors and Chromatin Accessibility in Hox Protein Target Specificity

Ching Yew Beh[1†], Sherif El-Sharnouby[2†], Aikaterini Chatzipli[23], Steven Russell[34], Siew Woh Choo[1*] and Robert White[2*]

[1]Department of Oral Biology and Biomedical Sciences, Faculty of Dentistry, University of Malaya, 50603 Kuala Lumpur, Malaysia
[2]Department of Physiology, Development and Neuroscience, University of Cambridge, Downing Street, Cambridge, CB2 3DY United Kingdom
[3]Department of Genetics, University of Cambridge, Downing Street, Cambridge, CB2 3EH United Kingdom
[4]Cambridge Systems Biology Centre, University of Cambridge, Tennis Court Road, Cambridge, CB2 1QR United Kingdom

[†]These authors contributed equally to this work.
*Corresponding authors: Siew Woh Choo and Robert White.

Ching Yew Beh behchingyew@gmail.com

Sherif El-Sharnouby bionouby@hotmail.com

Aikaterini Chatzipli chatzipli@gmail.com

Steven Russell sr120@hermes.cam.ac.uk

Siew Woh Choo lchoo@um.edu.my

Robert White rw108@cam.ac.uk

Additional File 2

Additional File 2 contains five supplementary figures.

Figure S4. Ubx wild type construct.

EcoRI + Kozak + eGFP (without stop codon) + KpnI + Ubx (with additional stop codon) + XbaI

GAATTCCAAAATGGTGAGCAAGGGCGAGGAGCTGTTCACCGGGGTGGTGCCCATCCTGGTCGAGCTGGACGG
CGACGTAAACGGCCACAAGTTCAGCGTGTCCGGCGAGGGCGAGGGCGATGCCACCTACGGCAAGCTGACCCT
GAAGTTCATCTGCACCACCGGCAAGCTGCCCGTGCCCTGGCCCACCCTCGTGACCACCCTGACCTACGGCGT
GCAGTGCTTCAGCCGCTACCCCGACCACATGAAGCAGCACGACTTCTTCAAGTCCGCCATGCCCGAAGGCTA
CGTCCAGGAGCGCACCATCTTCTTCAAGGACGACGGCAACTACAAGACCCGCGCCGAGGTGAAGTTCGAGGG
CGACACCCTGGTGAACCGCATCGAGCTGAAGGGCATCGACTTCAAGGAGGACGGCAACATCCTGGGGCACAA
GCTGGAGTACAACTACAACAGCCACAACGTCTATATCATGGCCGACAAGCAGAAGAACGGCATCAAGGTGAA
CTTCAAGATCCGCCACAACATCGAGGACGGCAGCGTGCAGCTCGCCGACCACTACCAGCAGAACACCCCCAT
CGGCGACGGCCCCGTGCTGCTGCCCGACAACCACTACCTGAGCACCCAGTCCGCCCTGAGCAAAGACCCCAA
CGAGAAGCGCGATCACATGGTCCTGCTGGAGTTCGTGACCGCCGCCGGGATCACTCTCGGCATGGACGAGCT
GTACAAGGGTACCATGAACTCGTACTTTGAACAGGCCTCCGGCTTTTATGGCCATCCGCACCAGGCCACCGG
AATGGCAATGGGCAGCGGTGGCCACCACGACCAGACGGCCAGTGCAGCGGCGGCCGCGTACAGAGGATTCCC
TCTCTCGCTGGGCATGAGTCCCTATGCCAACCACCATCTGCAGCGCACCACCCAGGACTCGCCCTACGATGC
CAGCATCACGGCCGCCTGCAACAAGATATACGGCGATGGAGCCGGAGCCTACAAACAGGACTGCCTGAACAT
CAAGGCGGATGCGGTGAATGGCTACAAAGACATTTGGAACACGGGCGGCTCGAATGGCGGCGGGGGTGGCGG
CGGAGGCGGTGGTGGCGGCGGAGCGGGCGGAACAGGTGGAGCCGGCAATGCCAATGGCGGTAATGCGGCCAA
TGCAAACGGACAGAACAATCCGGCGGGCGGTATGCCCGTTAGACCCTCCGCCTGCACCCCAGATTCCCGAGT
GGGCGGCTATTTGGACACGTCGGGCGGCAGTCCCGTTAGCCATCGCGGCGGCAGTGCCGGCGGTAATGTGAG
TGTCAGCGGCGGCAACGGCAACGCCGGAGGCGTACAGAGCGGCGTGGGCGTGGCCGGAGCGGGCACTGCCTG
GAATGCCAATTGCACCATCTCGGGCGCCGCTGCCCAAACGGCGGCCGCCAGCAGTTTACACCAGGCCAGCAA
TCACACATTCTACCCCTGGATGGCTATCGCAGGTAAGATAAGATCTGATTTAACACAATACGGCGGCATATC
AACAGACATGGGTAAGAGATACTCAGAATCTCTTGCGGGCTCACTTCTACCAGACTGGCTAGGTACAAATGG
TCTGCGAAGACGCGGCCGACAGACATACACCCGCTACCAGACGCTCGAGCTGGAGAAGGAGTTCCACACGAA
TCATTATCTGACCCGCAGACGGAGAATCGAGATGGCGCACGCGCTATGCCTGACGGAGCGGCAGATCAAGAT
CTGGTTCCAGAACCGGCGAATGAAGCTGAAGAAGGAGATCCAGGCGATCAAGGAGCTGAACGAACAGGAGAA
GCAGGCGCAGGCCCAGAAGGCGGCGGCGGCAGCGGCTGCGGCGGCGGCGGTCCAAGGTGGACACTTAGATCA
GTAGTAGTCTAGA

Hox homeodomain shown in grey, codons mutated to generate the Ubx mutant are underlined and mutated bases highlighted in yellow.

Figure S5. Abd-A construct.

EcoRI + Kozak + eGFP (without stop codon) + KpnI + Abd-A (with additional stop codon) + XbaI

GAATTCCAAAATGGTGAGCAAGGGCGAGGAGCTGTTCACCGGGGTGGTGCCCATCCTGGTCGAGCTGGACGG
CGACGTAAACGGCCACAAGTTCAGCGTGTCCGGCGAGGGCGAGGGCGATGCCACCTACGGCAAGCTGACCCT
GAAGTTCATCTGCACCACCGGCAAGCTGCCCGTGCCCTGGCCCACCCTCGTGACCACCCTGACCTACGGCGT
GCAGTGCTTCAGCCGCTACCCCGACCACATGAAGCAGCACGACTTCTTCAAGTCCGCCATGCCCGAAGGCTA
CGTCCAGGAGCGCACCATCTTCTTCAAGGACGACGGCAACTACAAGACCCGCGCCGAGGTGAAGTTCGAGGG
CGACACCCTGGTGAACCGCATCGAGCTGAAGGGCATCGACTTCAAGGAGGACGGCAACATCCTGGGGCACAA
GCTGGAGTACAACTACAACAGCCACAACGTCTATATCATGGCCGACAAGCAGAAGAACGGCATCAAGGTGAA
CTTCAAGATCCGCCACAACATCGAGGACGGCAGCGTGCAGCTCGCCGACCACTACCAGCAGAACACCCCCAT
CGGCGACGGCCCCGTGCTGCTGCCCGACAACCACTACCTGAGCACCCAGTCCGCCCTGAGCAAAGACCCCAA
CGAGAAGCGCGATCACATGGTCCTGCTGGAGTTCGTGACCGCCGCCGGGATCACTCTCGGCATGGACGAGCT
GTACAAGGGTACCATGTATCCGTACGTGTCTAACCACCCTAGCAGCCATGGAGGGCTCTCCGGAATGGCCGG
CTTCACCGGACTGGAGGATAAGTCGTGCAGCAGGTACACGGACACTGTCATGAACAGCTACCAGTCGATGAG
CGTACCTGCCTCGGCATCTGCACAGTTCGCTCAGTTCTATCAACATGCCACAGCCGCCGCATCCGCGGTATC
CGCGGCCAGTGCCGGCGCGATCGGCGTGGACTCGCTGGGAAATGCCTGCACACAGCCCGCCTCCGGCGTGAT
GCCAGGGGCAGGGGGAGCGGGCGGAGCCGGTATCGCCGATCTGCCCAGGTATCCCTGGATGACGCTTACAGA
CTGGATGGGAAGCCCCTTCGAGCGTGTCGTTTGTGGCGATTTCAACGGCCCCAACGGCTGTCCACGAAGGCG
CGGTCGCCAGACCTACACTCGCTTCCAGACCCTCGAACTGGAGAAGGAGTTTCACTTCAACCACTACTTAAC
TCGGCGAAGGCGCATCGAGATCGCACATGCCCTCTGCCTGACCGAGCGACAGATCAAGATCTGGTTTCAGAA
CCGTCGCATGAAGCTGAAGAAGGAGTTACGAGCCGTCAAGGAAATAAATGAACAGGCGCGACGCGATCGAGA
GGAGCAGGAGAAAATGAAGGCCCAGGAGACGATGAAATCCGCCCAGCAGAACAAGCAAGTGCAACAGCAGCA
GCAACAGCAACAGCAGCAGCAACAGCAGCAGCAGCAACAGCACCAACAGCAGCAACAACAGCCGCAGGACCA
CCACTCGATCATCGCACACAATCCAGGCCACTTGCACCACTCGGTGGTAGGTCAAAACGATCTCAAGCTCGG
CCTTGGCATGGGCGTGGGCGTCGGAGTGGGCGGCATCGGGCCGGGCATCGGTGGCGGCTTGGGCGGCAATCT
GGGCATGATGAGCGCCCTGGACAAGAGCAATCACGACCTGCTAAAGGCGGTCAGCAAAGTCAACTCCTAATA
ATCTAGA

Hox homeodomain shown in grey.

Figure S6. Abd-B construct.

EcoRI + Kozak + eGFP (without stop codon) + KpnI + Abd-B (with additional stop codon) + XbaI

```
GAATTCCAAAATGGTGAGCAAGGGCGAGGAGCTGTTCACCGGGGTGGTGCCCATCCTGGTCGAGCTGGACGG
CGACGTAAACGGCCACAAGTTCAGCGTGTCCGGCGAGGGCGAGGGCGATGCCACCTACGGCAAGCTGACCCT
GAAGTTCATCTGCACCACCGGCAAGCTGCCCGTGCCCTGGCCCACCCTCGTGACCACCCTGACCTACGGCGT
GCAGTGCTTCAGCCGCTACCCCGACCACATGAAGCAGCACGACTTCTTCAAGTCCGCCATGCCCGAAGGCTA
CGTCCAGGAGCGCACCATCTTCTTCAAGGACGACGGCAACTACAAGACCCGCGCCGAGGTGAAGTTCGAGGG
CGACACCCTGGTGAACCGCATCGAGCTGAAGGGCATCGACTTCAAGGAGGACGGCAACATCCTGGGGCACAA
GCTGGAGTACAACTACAACAGCCACAACGTCTATATCATGGCCGACAAGCAGAAGAACGGCATCAAGGTGAA
CTTCAAGATCCGCCACAACATCGAGGACGGCAGCGTGCAGCTCGCCGACCACTACCAGCAGAACACCCCCAT
CGGCGACGGCCCCGTGCTGCTGCCCGACAACCACTACCTGAGCACCCAGTCCGCCCTGAGCAAAGACCCCAA
CGAGAAGCGCGATCACATGGTCCTGCTGGAGTTCGTGACCGCCGCCGGGATCACTCTCGGCATGGACGAGCT
GTACAAGGGTACCATGTCCATACAATTAGCGCCACTGCATATACCCGCCATCCGGGCCGGTCCGGGATTCGA
GACGGACACCTCGGCGGCGGTCAAGCGGCACACGGCACACTGGGCCTACAACGACGAGGGATTCAATCAGCA
TTACGGCTCCGGGTACTACGACCGCAAGCACATGTTCGCCTATCCTTACCCAGAAACGCAGTTTCCGGTTGG
TCAGTACTGGGGCCCCAACTACCGCCCCGATCAGACCACCTCTGCCGCAGCGGCGGCGGCCTACATGAACGA
AGCGGAGCGCCACGTGAGCGCCGCCGCCCGACAGTCCGTCGAGGGCACATCGACGTCCAGCTATGAGCCGCC
CACCTACTCCTCGCCAGGCGGCCTGCGCGGCTATCCCAGCGAGAACTACTCCAGCTCAGGAGCCTCTGGTGG
ATTATCCGTGGGAGCAGTGGGTCCTTGCACGCCCAATCCCGGACTGCACGAGTGGACCGGTCAGGTGTCCGT
CCGGAAAAAGCGCAAGCCGTACTCGAAGTTCCAGACCCTGGAGCTGGAGAAGGAGTTTCTTTTCAATGCGTA
TGTTTCCAAGCAAAAGCGCTGGGAATTGGCCAGAAATTTGCAGCTGACCGAGCGACAGGTCAAGATATGGTT
CCAGAATCGGCGCATGAAGAACAAGAAGAACTCACAGCGCCAGGCCAATCAGCAGAACAACAACAACAATTC
GAGCAGCAACCACAACCACGCGCAGGCGACCCAGCAGCACCACAGCGGCCACCACCTGAACCTTAGCCTGAA
CATGGGTCACCATGCCGCCAAGATGCACCAGTGATGATCTAGA
```

Hox homeodomain shown in grey, the A reintroduced to rectify the point mutation in the cDNA sequence is highlighted in yellow [the Abd-B clone had a single point deletion of an A at position 1289 (position 578 of the CDS), which was reintroduced by site-directed mutagenesis].

Figure S7. Ubx mutant construct.

EcoRI + Kozak + eGFP (without stop codon) + KpnI + Ubx (with additional stop codon) + XbaI

GAATTCCAAAATGGTGAGCAAGGGCGAGGAGCTGTTCACCGGGGTGGTGCCCATCCTGGTCGAGCTGGACGG
CGACGTAAACGGCCACAAGTTCAGCGTGTCCGGCGAGGGCGAGGGCGATGCCACCTACGGCAAGCTGACCCT
GAAGTTCATCTGCACCACCGGCAAGCTGCCCGTGCCCTGGCCCACCCTCGTGACCACCCTGACCTACGGCGT
GCAGTGCTTCAGCCGCTACCCCGACCACATGAAGCAGCACGACTTCTTCAAGTCCGCCATGCCCGAAGGCTA
CGTCCAGGAGCGCACCATCTTCTTCAAGGACGACGGCAACTACAAGACCCGCGCCGAGGTGAAGTTCGAGGG
CGACACCCTGGTGAACCGCATCGAGCTGAAGGGCATCGACTTCAAGGAGGACGGCAACATCCTGGGGCACAA
GCTGGAGTACAACTACAACAGCCACAACGTCTATATCATGGCCGACAAGCAGAAGAACGGCATCAAGGTGAA
CTTCAAGATCCGCCACAACATCGAGGACGGCAGCGTGCAGCTCGCCGACCACTACCAGCAGAACACCCCCAT
CGGCGACGGCCCCGTGCTGCTGCCCGACAACCACTACCTGAGCACCCAGTCCGCCCTGAGCAAAGACCCCAA
CGAGAAGCGCGATCACATGGTCCTGCTGGAGTTCGTGACCGCCGCCGGGATCACTCTCGGCATGGACGAGCT
GTACAAGGGTACCATGAACTCGTACTTTGAACAGGCCTCCGGCTTTTATGGCCATCCGCACCAGGCCACCGG
AATGGCAATGGGCAGCGGTGGCCACCACGACCAGACGGCCAGTGCAGCGGCGGCCGCGTACAGAGGATTCCC
TCTCTCGCTGGGCATGAGTCCCTATGCCAACCACCATCTGCAGCGCACCACCCAGGACTCGCCCTACGATGC
CAGCATCACGGCCGCCTGCAACAAGATATACGGCGATGGAGCCGGAGCCTACAAACAGGACTGCCTGAACAT
CAAGGCGGATGCGGTGAATGGCTACAAAGACATTTGGAACACGGGCGGCTCGAATGGCGGCGGGGGTGGCGG
CGGAGGCGGTGGTGGCGGCGGAGCGGGCGGAACAGGTGGAGCCGGCAATGCCAATGGCGGTAATGCGGCCAA
TGCAAACGGACAGAACAATCCGGCGGGCGGTATGCCCGTTAGACCCTCCGCCTGCACCCCAGATTCCCGAGT
GGGCGGCTATTTGGACACGTCGGGCGGCAGTCCCGTTAGCCATCGCGGCGGCAGTGCCGGCGGTAATGTGAG
TGTCAGCGGCGGCAACGGCAACGCCGGAGGCGTACAGAGCGGCGTGGGCGTGGCCGGAGCGGGCACTGCCTG
GAATGCCAATTGCACCATCTCGGGCGCCGCTGCCCAAACGGCGGCCGCCAGCAGTTTACACCAGGCCAGCAA
TCACACATTCTACCCCTGGATGGCTATCGCAGGTAAGATAAGATCTGATTTAACACAATACGGCGGCATATC
AACAGACATGGGTAAGAGATACTCAGAATCTCTTGCGGGCTCACTTCTACCAGACTGGCTAGGTACAAATGG
TCTGCGAAGAGCCGGCGCACAGACATACACCCGCTACCAGACGCTCGAGCTGGAGAAGGAGTTCCACACGAA
TCATTATCTGACCCGCAGACGGAGAATCGAGATGGCGCACGCGCTATGCCTGACGGAGCGGCAGATCAAGGC
CTGGTTCAAGGCCCGGCGAATGAAGCTGAAGAAGGAGATCCAGGCGATCAAGGAGCTGAACGAACAGGAGAA
GCAGGCGCAGGCCCAGAAGGCGGCGGCGGCAGCGGCTGCGGCGGCGGCGGTCCAAGGTGGACACTTAGATCA
GTAGTAGTCTAGA

Hox homeodomain shown in grey, mutated codons underlined, mutated bases highlighted in yellow.

Figure S8. Ubx+Hth construct.

EcoRI + Kozak + Hth (without stop codon) + NotI + T2A + XbaI + eGFP (without stop codon) + KpnI + Ubx (with additional stop codon) + ApaI

GAATTCCAAAATGGCTCAGCCCAGGTATGACGATGGCCTACACGGCTACGGCATGGACTCCGGAGCCGCAGC
AGCGGCCATGTACGATCCACACGCCGGTCACCGGCCGCCCGGACTGCAGGGCCTCCCCTCGCACCACTCTCC
GCACATGACGCACGCAGCGGCGGCGGCGGCCACAGTGGGCATGCACGGCTACCATTCGGGGGCCGGGGGTCA
TGGAACACCTAGTCATGTATCGCCGGTCGGTAATCACCTAATGGGCGCAATACCCGAAGTACACAAACGTGA
TAAGGATGCGATTTATGAACATCCGCTATTCCCGCTTTTGGCGCTGATCTTTGAGAAGTGCGAATTGGCCAC
ATGTACGCCGAGGGAGCCCGGTGTGCAAGGTGGCGATGTCTGTTCGTCGGAATCGTTCAACGAGGATATTGC
AATGTTCAGTAAACAGATAAGATCACAGAAACCCTATTATACCGCAGATCCCGAAGTCGACTCACTGATGGT
GCAAGCAATACAAGTACTTCGGTTTCACCTTTTAGAATTAGAAAAAGTACACGAGTTATGCGATAACTTCTG
TCATCGGTATATATCGTGTTTAAAGGGTAAAATGCCAATAGATTTAGTGATCGACGAACGGGACACCACCAA
ACCACCGGAGTTGGGATCGGCGAACGGAGAAGGGCGCAGCAACGCCGACTCCACATCGCACACCGATGGAGC
TAGTACACCAGACGTTCGGCCGCCGAGCTCATCGCTGTCCTACGGCGGCGCAATGAACGATGACGCCCGATC
GCCGGGCGCTGGTAGTACTCCCGGTCCACTGTCACAGCAGCCACCTGCCCTAGATACATCAGACCCTGATGG
ACGATGGTGTCGAAGAGAATGGTCCTCACCTGCCGATGCTCGCAATGCAGACGCTTCCCGGCGATTGTATTC
CTCAGTCTTCCTCGGTAGTCCTGACAATTTCGGAACGAGTGCAAGCGGTGATGCCAGCAATGCCAGCATAGG
AAGTGGAGAGGGCACCGGCGAGGAGGACGACGATGCGAGCGGCAAAAAGAACCAAAAGAAACGTGGCATTTT
CCCAAAAGTAGCAACCAACATATTGAGAGCGTGGCTGTTTCAGCATTTAACGCATCCCTACCCATCCGAGGA
CCAGAAGAAACAATTGGCCCAGGACACCGGCCTAACGATACTGCAAGTGAATAATTGGTTCATCAATGCGCG
GCGGAGAATTGTCCAGCCAATGATCGATCAATCGAATCGTGCAGTCTATACACCGCATCCAGGTCCCTCCGG
ATATGGCCACGACGCCATGGGCTACATGATGGACAGCCAGGCGCATATGATGCACCGTCCGCCCGGAGATCC
GGGCTTCCACCAGGGCTATCCGCATTACCCGCCCGCCGAGTACTACGGCCAGCACTTGGCGGCCGCCGAGGG
CAGAGGAAGTCTTCTAACATGCGGTGACGTGGAGGAGAATCCCGGCCCTTCTAGAATGGTGAGCAAGGGCGA
GGAGCTGTTCACCGGGGTGGTGCCCATCCTGGTCGAGCTGGACGGCGACGTAAACGGCCACAAGTTCAGCGT
GTCCGGCGAGGGCGAGGGCGATGCCACCTACGGCAAGCTGACCCTGAAGTTCATCTGCACCACCGGCAAGCT
GCCCGTGCCCTGGCCCACCCTCGTGACCACCCTGACCTACGGCGTGCAGTGCTTCAGCCGCTACCCCGACCA
CATGAAGCAGCACGACTTCTTCAAGTCCGCCATGCCCGAAGGCTACGTCCAGGAGCGCACCATCTTCTTCAA
GGACGACGGCAACTACAAGACCCGCGCCGAGGTGAAGTTCGAGGGCGACACCCTGGTGAACCGCATCGAGCT
GAAGGGCATCGACTTCAAGGAGGACGGCAACATCCTGGGGCACAAGCTGGAGTACAACTACAACAGCCACAA
CGTCTATATCATGGCCGACAAGCAGAAGAACGGCATCAAGGTGAACTTCAAGATCCGCCACAACATCGAGGA
CGGCAGCGTGCAGCTCGCCGACCACTACCAGCAGAACACCCCCATCGGCGACGGCCCCGTGCTGCTGCCCGA
CAACCACTACCTGAGCACCCAGTCCGCCCTGAGCAAAGACCCCAACGAGAAGCGCGATCACATGGTCCTGCT
GGAGTTCGTGACCGCCGCCGGGATCACTCTCGGCATGGACGAGCTGTACAAGGGTACCATGAACTCGTACTT
TGAACAGGCCTCCGGCTTTTATGGCCATCCGCACCAGGCCACCGGAATGGCAATGGGCAGCGGTGGCCACCA
CGACCAGACGGCCAGTGCAGCGGCGGCCGCGTACAGAGGATTCCCTCTCTCGCTGGGCATGAGTCCCTATGC
CAACCACCATCTGCAGCGCACCACCCAGGACTCGCCCTACGATGCCAGCATCACGGCCGCCTGCAACAAGAT
ATACGGCGATGGAGCCGGAGCCTACAAACAGGACTGCCTGAACATCAAGGCGGATGCGGTGAATGGCTACAA
AGACATTTGGAACACGGGCGGCTCGAATGGCGGCGGGGGTGGCGGCGGAGGCGGTGGTGGCGGCGGAGCGGG
CGGAACAGGTGGAGCCGGCAATGCCAATGGCGGTAATGCGGCCAATGCAAACGGACAGAACAATCCGGCGGG
CGGTATGCCCGTTAGACCCTCCGCCTGCACCCCAGATTCCCGAGTGGGCGGCTATTTGGACACGTCGGGCGG
CAGTCCCGTTAGCCATCGCGGCGGCAGTGCCGGCGGTAATGTGAGTGTCAGCGGCGGCAACGGCAACGCCGG
AGGCGTACAGAGCGGCGTGGGCGTGGCCGGAGCGGGCACTGCCTGGAATGCCAATTGCACCATCTCGGGCGC
CGCTGCCCAAACGGCGGCCGCCAGCAGTTTACACCAGGCCAGCAATCACACATTCTACCCCTGGATGGCTAT
CGCAGGTAAGATAAGATCTGATTTAACACAATACGGCGGCATATCAACAGACATGGGTAAGAGATACTCAGA
ATCTCTTGCGGGCTCACTTCTACCAGACTGGCTAGGTACAAATGGTCTGCGAAGACGCGGCCGACAGACATA
CACCCGCTACCAGACGCTCGAGCTGGAGAAGGAGTTCCACACGAATCATTATCTGACCCGCAGACGGAGAAT
CGAGATGGCGCACGCGCTATGCCTGACGGAGCGGCAGATCAAGATCTGGTTCCAGAACCGGCGAATGAAGCT
GAAGAAGGAGATCCAGGCGATCAAGGAGCTGAACGAACAGGAGAAGCAGGCGCAGGCCCAGAAGGCGGCGGC
GGCAGCGGCTGCGGCGGCGGCGGTCCAAGGTGGACACTTAGATCAGTAGTAGGGGCCC

Homeodomains shown in grey, additional base added to maintain the correct reading frame highlighted in yellow, the amino acids added to the Ubx-GFP protein after 2A-mediated cleavage are underlined.
